# Mast Cells Participate in Corneal Development in Mice

**DOI:** 10.1038/srep17569

**Published:** 2015-12-02

**Authors:** Jun Liu, Ting Fu, Fang Song, Yunxia Xue, Chaoyong Xia, Peng Liu, Hanqing Wang, Jiajun Zhong, Quanrong Li, Jiansu Chen, Yangqiu Li, Dongqing Cai, Zhijie Li

**Affiliations:** 1 Key Laboratory for Regenerative Medicine, Ministry of Education, Jinan University, Guangzhou, China; 2 International Collaborative Innovation Research Center of Ocular Surface Diseases and Institute of Ophthalmology, Jinan University School of Medicine, Guangzhou, China; 3 Department of Embryology and Histology, Jinan University School of Medicine, Guangzhou, China; 4 Institute of Hematology, Jinan University School of Medicine, Guangzhou, China; 5 Department of Ophthalmology, First Affiliated Hospital of Jinan University, Guangzhou, China; 6 Section of Leukocyte Biology, Department of Pediatrics, Children’s Nutrition Research Center, Baylor College of Medicine, Houston, Texas, USA

## Abstract

The development of the cornea, a highly specialized transparent tissue located at the anterior of the eye, is coordinated by a variety of molecules and cells. Here, we report that mast cells (MCs), recently found to be involved in morphogenesis, played a potentially important role in corneal development in mice. We show that two different waves of MC migration occurred during corneal development. In the first wave, MCs migrated to the corneal stroma and became distributed throughout the cornea. This wave occurred by embryonic day 12.5, with MCs disappearing from the cornea at the time of eyelid opening. In the second wave, MCs migrated to the corneal limbus and became distributed around limbal blood vessels. The number of MCs in this region gradually increased after birth and peaked at the time of eyelid opening in mice, remaining stable after postnatal day 21. We also show that integrin α4β7 and CXCR2 were important for the migration of MC precursors to the corneal limbus and that c-Kit-dependent MCs appeared to be involved in the formation of limbal blood vessels and corneal nerve fibers. These data clearly revealed that MCs participate in the development of the murine cornea.

The eye is a very complex organ. It is composed of three major tissues: the cornea, the lens, and the retina. The cornea and lens gather, focus, and transmit light to form an image of the outside world on the retina. The retina converts the image into neural signals and sends these signals to the brain for visual recognition. The formation and development of the eye depends on highly organized processes. Defects in cell lineage of various origins and mutations in key genes lead to severe, congenital disorders. Aside from leading to visual impairment, loss of eye function may cause social and economic handicaps and can even lead to personality changes. Thus, characterization of the development of ocular tissues during embryogenesis is important to understand the pathogenesis of congenital anomalies of the eye^1^ and may eventually lead to the treatment of these anomalies.

The cornea is a highly specialized transparent tissue located at the anterior of the eye, providing more than two-thirds of the total refractive power of the eye. Thus, maintenance of normal structure and transparency is critical for normal light refraction. The cornea is composed of the anterior epithelium, central stroma, and posterior endothelium. During development in mice, the morphogenesis of the cornea involves two orderly cellular migration and differentiation events. First, at around embryonic day (E) 11.5, the surface ectoderm differentiates into the presumptive corneal epithelium. Second, around E12.5, periocular mesenchymal cells migrate into the region between the lens vesicle and the developing corneal epithelium to give rise to the corneal endothelium and stromal keratocytes. The entire process is coordinated by a complex network of molecules and cells^2^,^3^.

The tissues are formed by the periocular mesenchymal cells that have well-established roles in the formation of the corneal stroma and endothelium^2^. However, the roles in corneal development of other cells in the stroma have not yet been determined. Immune cells, some of which are known to participate in organogenesis via different mechanisms and signaling pathways^4^,^5^,^6^, may be present in the mesenchymal layers of the developing eye. Macrophages are well-studied immune cells that function in the development of many tissues. Their absence during development causes remodeling defects in various tissues, including the mammary gland, kidney, and pancreas, suggesting a general requirement for macrophages in tissue-patterning and -branching morphogenesis^4^. The hyaloid vasculature, which is a complex system of transient intraocular vessels, regresses during the second month of human gestation. Early in postnatal mouse development, during the regression of the hyaloid vasculature, macrophages identify vascular endothelial cells and instruct them to undergo apoptosis^5^. However, the function of other immune cells in organogenesis has been less studied.

Mast cells (MCs) are innate immune cells and are generally considered to originate from a multipotent hematopoietic progenitor in the bone marrow. They partially differentiate in bone marrow and enter the circulation as committed MC progenitors. In the circulation and under the influence of selective chemotactic and growth factors, they are selectively attracted to peripheral tissues, where they terminally mature into cells with different phenotypes and granule contents^6^,^7^,^8^,^9^. However, studies have demonstrated that MC precursors are also found in the yolk sac at E9.5^10^ and subsequently increase in number from E11 to E15 in the fetal liver^11^. MCs can be activated by diverse stimuli to release their pre-packaged granules, which contain bioactive molecules, such as histamine, serine proteases, pro-inflammatory cytokines, pro-angiogenic factors, and chemokines. Following MC degranulation, these mediators can contribute to allergic reactions, protection against parasitic or bacterial infections, and the promotion of wound healing^12^. Recent evidence has suggested that MCs are involved in the morphogenesis of certain mouse organs, such as the mammary glands^13^. Despite these novel observations, the role of MCs in the morphogenesis of the mouse cornea remains unexplored.

To address this issue, we examined the distribution of MCs in mouse corneas and mechanisms that control the development of MCs in the cornea that are important to understand the roles of these cells in corneal morphogenesis. Subsequently, we observed the potential effects of MCs on the morphogenesis of the cornea, particularly focusing on the formation of the limbal vascular network and innervation of the cornea, using c-Kit-dependent, MC-deficient mice.

## Results

### MCs were identified in the corneal limbus of adult mice

Avidin binds specifically to intracellular heparin in the MC cytoplasm, and avidin staining is currently regarded as a reliable and convenient method for identifying rodent and human MCs^14^,^15^,^16^. Avidin-stained cells were found in the corneal limbus and conjunctival parenchyma but not in the central cornea of adult mice (Supplementary Fig. 1A). To confirm that these cells in the corneal limbus were MCs, they were co-stained with avidin (Supplementary Fig. 1B, left) and Toluidine Blue O (Supplementary Fig. 1B, center), a dye classically used to label MCs. The merged image (Supplementary Fig. 1B, right) shows the co-localization of the two stains. Under high-resolution immunofluorescence microscopy, the avidin-positive cells in the adult corneal limbus were seen to contain the intracellular micro-particles that are typical of MCs (Supplementary Fig. 1C, left). Under transmission electron microscopy, corneal limbus had some cells with the ultrastructure that was typical of MCs. Many granules with highly electron-dense content were visible in the cells (Supplementary Fig. 1C, center and right). Flow cytometry analysis revealed that avidin-positive cells were both CD45 and cKit positive (Supplementary Fig. 1D). CD45 is a common marker of all hematopoietic cells, and cKit constitutively expresses in MCs^17^. Therefore, the avidin-positive cells observed in our study are MCs. Finally, the limbal MCs stained red (Supplementary Fig. 1E) when stained with Alcian Blue and Safranin O, which are used to differentiate between the maturation states of MCs. Immature MCs are known to turn blue when stained with Alcian Blue and Safranin O, whereas mature MCs turn red^18^.

### MCs localized to the central stroma in the first wave of migration

**Figure 1:**
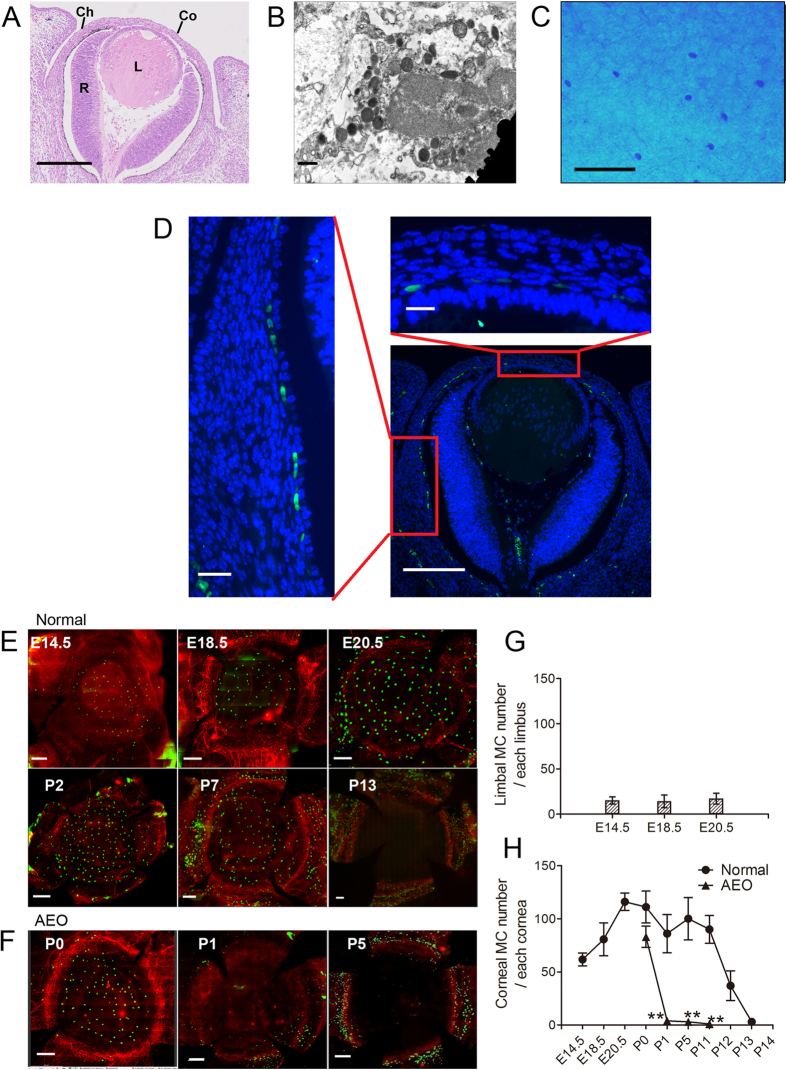
Features and dynamic changes of the first wave MCs during embryonic and neonatal period. (**A**) This panel shows hematoxylin and eosin staining of a paraffin section taken from an E12.5 mouse eye. The eyelid is not closed at this time. Co, cornea; L, lens; R, retina; and Ch, choroid. Scale bar: 100 μm. (**B**) This panel contains a transmission electron microscopy image of MCs from an E14.5 mouse cornea. The cytoplasmic granules in this cell are small, heterogeneous, and less electron-dense. Scale bar: 0.5 μm. (**C**) This panel depicts Alcian Blue–Safranin O staining of an E14.5 mouse cornea. Only Alcian Blue-stained cells are detected. Scale bar: 100 μm. (**D**) This panel shows immunostaining with avidin-FITC (green) and DAPI (blue) of an eyeball section from an E12.5 mouse. Avidin-positive cells are observed among the posterior mesenchymal cells (upper-right image), and appeared to be continuous with the MCs in the presumptive choroid stroma (left image). Scale bars: lower-right image, 100 μm; upper-right and left images, 20 μm. (**E**) These images depict immunostaining of cornea with complete limbus with CD31 PE (red) and avidin-FITC (green) during different developmental stages from E14.5 to P13. Scale bars: 200 μm. (**F**) These panels show immunostaining of cornea with complete limbus from AEO mice with CD31 PE and avidin-FITC at the developmental time points indicated. Scale bars: 200 μm. (**G**) The number of MCs that migrate into the limbus during the embryonic stages are indicated in this graph. Across these developmental stages, limbal MC number remained relatively unchanged (n = 6 corneas per age group). (**H**) This graph depicts the number of MCs that migrated into the corneas of normal and AEO mice during the developmental stages indicated. The number of corneal MCs in normal mice increased from E14.5 to E20, gradually decreased after birth, and disappeared at P13. The number of corneal MCs in AEO mice disappeared at P1 (n = 6 corneas per age group). **P < 0.01.

At E12.5, the ectoderm detaches from the lens, leaving a space. Mesenchymal cells start to migrate into this space, forming the future corneal stroma and endothelium^2^,^3^. To investigate the distribution of MCs in the embryonic cornea, we stained eye sections from E12.5 mice. Hematoxylin and eosin staining of paraffin sections revealed the structure of the eye in E12.5 mice. The eye at this time already had a presumptive cornea with several stromal cell layers (Fig. 1A). MCs were already present in the presumptive corneal stroma at E12.5 and were located primarily among posterior mesenchymal cells (Fig. 1D, right images). MCs were also seen in the choroid and primitive vitreous chamber between the lens and retina. MCs in the presumptive cornea appeared to be continuous with those MCs in the presumptive choroid stroma (Fig. 1D, left image). Thus, MCs may accompany periocular mesenchymal cells as they migrate into the ocular tissue, including into the corneal stroma. Whole-mount immunostaining of E14.5 mouse corneas shows that this first wave of MC migration localized primarily to the cornea rather than to the limbus (Fig. 1E, top left image). We designated MCs in the cornea as corneal MCs and those around the limbal blood vessels as limbal MCs. To investigate the dynamic change of MC migration before and after birth, six corneas with complete limbus obtained from mice of specified ages were stained with avidin-fluorescein isothiocyanate (FITC) and anti-CD31 phycoerythrin (PE) antibodies. The number of limbal MCs remained relatively constant, but the number of corneal MCs gradually increased from E14.5 to E20.5 (Fig. 1E, top three images). This first wave of corneal MCs gradually decreased after birth, from postnatal day (P) 0, and rapidly disappeared by P13, which is the time at which mouse eyelids open (Fig. 1E, bottom three images). Rapid disappearance of these cells may be a consequence of the ocular surface exposure to the outside environment after eyelid opening. To test this hypothesis, we surgically opened mouse eyelids at P0. As expected, the first wave of corneal MCs in mice that underwent artificial eye opening (AEO) disappeared the next day (Fig. 1F). These data suggest that eyelid opening influences the distribution of MCs in the cornea. The dynamic changes are reflected in the number of avidin-positive MCs at various stages from E14.5 to P13 (Fig. 1G,H).

### MCs localized to the limbal vasculature in a second wave of postnatal migration

**Figure 2:**
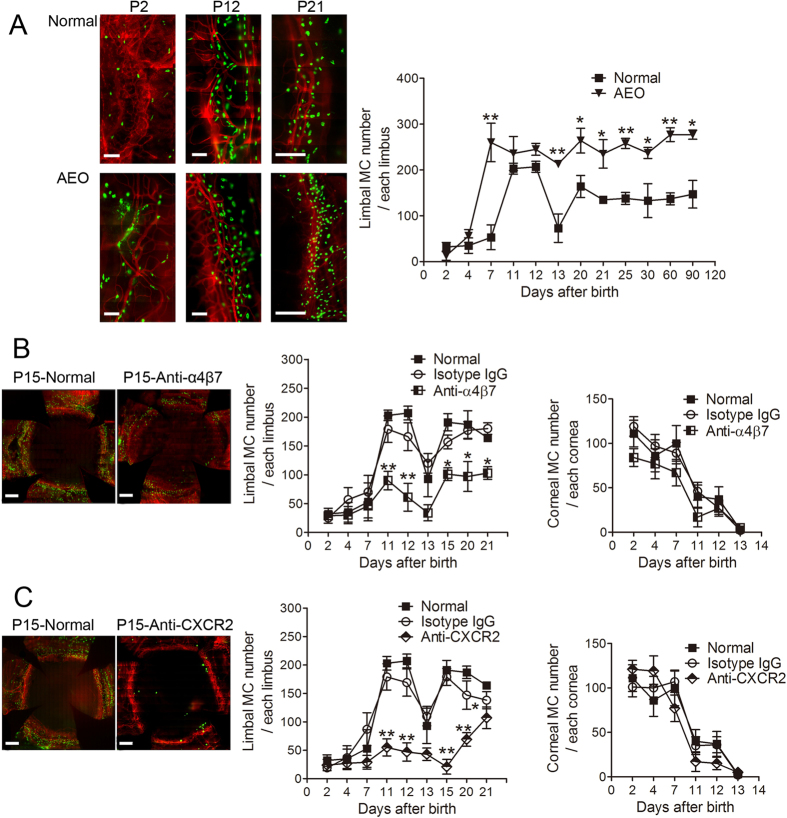
Dynamic changes in the second wave of MC migration and the effect of blocking antibodies. (**A**) These panels show the second wave of MC migration. These left images depict avidin-FITC (green) and CD31 PE (red) immunostaining of the corneal limbus from normal mice and from AEO mice at P2, P12, and P21. Scale bars: 200 μm. The graph depicts the dynamic changes of the second-wave MC in the limbus from normal and AEO mice during development (n = 6 corneas per age group). *P < 0.05; **P < 0.01. (**B**) These panels show the two waves of limbal and corneal MC migration in normal mice and after injection with an anti-integrin-α4β7 antibody (with isotype IgG injection as control). Immunostaining with CD31 PE (red) and avidin-FITC (green) of corneas with complete limbus from normal and anti-α4β7-treated mice are shown in the left two images. Scale bars: 400 μm. The two graphs depict the dynamic changes of the two waves of MCs distributed in the limbus and cornea, respectively, after anti-α4β7 antibody and isotype IgG injection (n = 6 corneas per age group). *P < 0.05; **P < 0.01. (**C**) The two waves of limbal and corneal MC migration in normal mice and after injection with an anti-CXCR2 antibody (with isotype IgG control) are shown in these panels. The left two images show immunostaining of corneas with complete limbus from normal and anti-CXCR2 treated mice. Scale bars: 400 μm. The graphs depict the dynamic changes of the two waves of MCs distributed in the limbus and cornea, respectively, after anti-CXCR2 antibody and isotype IgG injection (n = 6 corneas per age group). *P < 0.05; **P < 0.01.

As the number of corneal MCs gradually decreased during the early neonatal period, MCs accumulated around the corneal vasculature. Although there were few MCs around the corneal limbal blood vessels at P1, the number of MCs gradually increased, peaking at P11-12. Limbal MCs decreased in number at P13 but then stabilized and remained stable after P21 (Fig. 2A). The developmental pattern of the Limbal MCs was also altered by AEO at P0, which caused the cell number to peak earlier at P7. Strikingly, in mice that underwent AEO (AEO mice), the total number of MCs remained high for at least 3 months (Fig. 2A). The first-wave MCs were ultra-structurally immature with small, heterogeneous, and less electron-dense cytoplasmic granules (Fig. 1B). By contrast, the second-wave MCs were mature with relatively homogeneous, more electron-dense granules (Supplementary Fig. 1C, middle and right). Moreover, whereas the first-wave MCs stained blue when stained with Alcian Blue and Safranin O (Fig. 1C), the second wave MCs stained red (Supplementary Fig. 1E). Thus, the first-wave is likely to comprise immature MCs and the second-wave mature MCs.

### Integrin α4β7 and CXCR2 were involved in MC migration to the corneal limbus

MC precursors are found in various peripheral organs^19^. Studies of the small intestine and inflamed lung tissues have indicated that the recruitment of MC progenitors to tissues is highly controlled by different integrins and chemokines^20^,^21^,^22^. These studies indicate interactions between integrin α4β7 of MC progenitors and its ligand, mucosal vascular addressin cell adhesion molecule 1 (MAdCAM-1), which is present on the mucosal microvasculature^20^, are critical for the localization of MCs to intestinal tissue. The expression of the chemokine receptor CXCR2 by circulating MC precursors is also required for their localization to the small intestine^23^. Integrin α4β7 and its other ligand, vascular cell adhesion molecule (VCAM-1), are required for the recruitment of MC precursors to the lung^21^. CXCR2 expression by lung cells regulates VCAM-1 expression on the lung endothelium and is required for the antigen-induced recruitment of MC progenitors into the lung^24^. To assess the roles of integrin α4β7 and CXCR2 in the migration of MCs, neonatal mice were treated with anti-α4β7 or anti-CXCR2 antibodies. Treatment with either antibody blocked the migration of MCs to the corneal limbus. However, treatment with these antibodies did not affect the number of first-wave MCs after birth, which followed their normal developmental patterns and disappeared by P13 (Fig. 2B,C).

### The expression levels of related migration and proliferation factors in the cornea were changed during development

**Figure 3:**
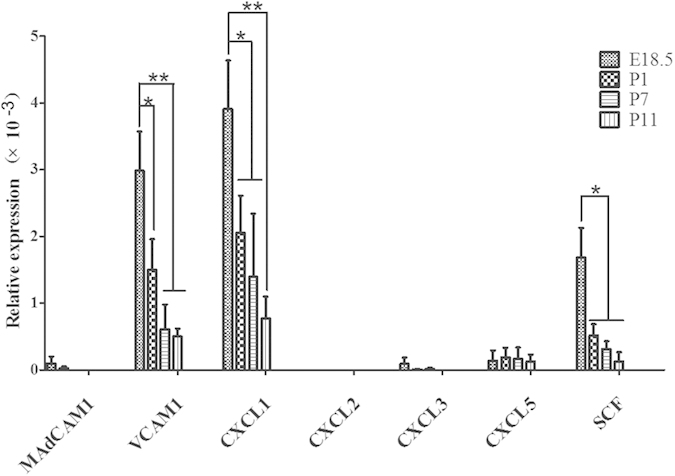
Relative gene expression levels of MC migration-related factors in the corneas of embryonic and postnatal mice. The expression of the following factors was assayed by RT-PCR: MAdCAM1, CXCL1, CXCL2, CXCL3, CXCL5, VCAM1, and SCF at the developmental time points shown above. MAdCAM1, CXCL2, CXCL3, and CXCL5 were expressed at low levels or not expressed in the corneal tissue assayed. The expression of VCAM1, CXCL1, and SCF decreased after birth (n = 3 repeat experiments, each experiment: E18.5-P11, 20 corneas). The relative expression of the target genes were compared with GAPDH. *P < 0.05; **P < 0.01.

Given that integrin α4β7 and CXCR2 are required for the migration of MC precursors to the cornea, we assessed the expression of their ligands in the cornea as well as that of related adhesion molecules and chemokines. Integrin α4β7 can bind to both ligands MAdCAM-1 and VCAM-1^23^,^25^. CXCL1, CXCL2, CXCL3, and CXCL5 are the primary ligands of mouse CXCR2^26^. Thus, we investigated the expression of these six molecules during corneal development as possible indicators of MC migration to the cornea. Stem cell factor (SCF) is also involved in the development of MCs and induces their proliferation and maturation as well as their production of heparin^27^. Expression of this factor in the cornea could affect the distribution of MCs in the cornea. Real-time quantitative polymerase chain reaction (RT-PCR) results indicated that MAdCAM1, CXCL2, CXCL3, and CXCL5 were either expressed at low levels or not expressed in the cornea, whereas VCAM1, CXCL1, and SCF were substantially expressed (Fig. 3). Thus, in corneal development, the primary ligand for integrin α4β7 is VCAM1, and the primary ligand for CXCR2 is CXCL1. The expression of VCAM1, CXCL1, and SCF decreased sharply after birth (Fig. 3), indicating that the disappearance of MCs from the cornea may be related to the down-regulation of these molecules in corneal tissue.

### Corneal and limbal MCs were capable of proliferation during eye development

**Figure 4:**
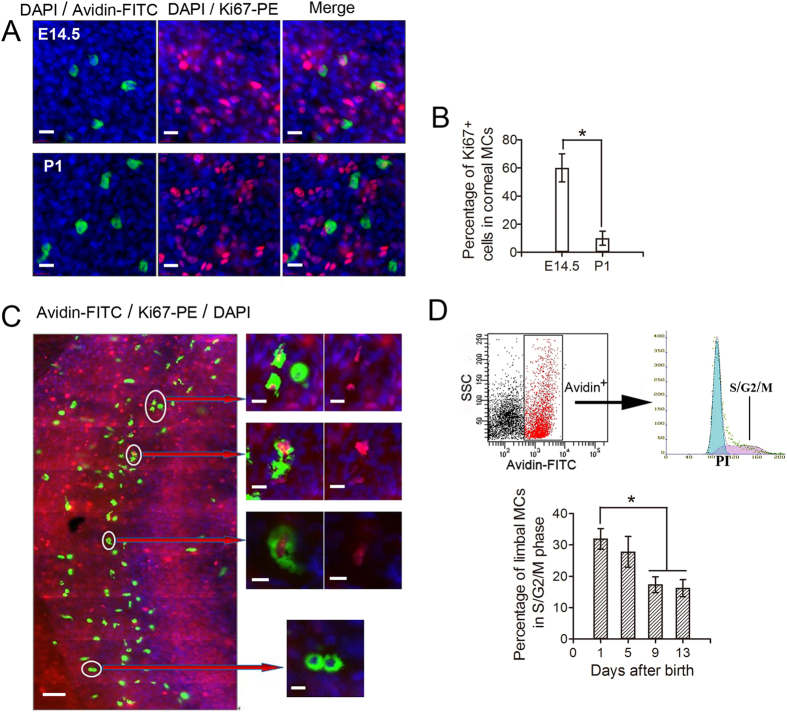
Proliferation of MCs. (**A**) Co-staining of corneas from E14.5 and P1 mice with anti-mouse Ki67-PE (red), avidin-FITC (green), and DAPI (blue) are depicted in these images. Avidin-positive MCs in the E14.5 mouse cornea were almost all positive for Ki67 (top three images), whereas avidin and Ki67 staining co-localized in just a few MCs in the P1 mouse cornea (bottom three images). Scale bars: 20 μm. (**B**) The histogram shows the percentages of Ki67-positive corneal MCs. A significantly smaller percentage of Ki67-positive MCs was detected in the P1 mouse cornea compared to the E14.5 mouse cornea (n = 6 corneas per age group). *P < 0.05. (**C**) Co-staining of the corneal limbi from P30 mice with anti-mouse Ki67-PE (red), avidin-FITC (green), and DAPI (blue) is shown. Some MCs were positive for both avidin and Ki67 (right, top six images), and some were undergoing mitosis (right, bottom image). Scale bars: left image, 100 μm; magnified right images, 20 μm. (**D**) Cell-cycle analysis of second-wave MCs by flow cytometry is shown. The top image depicts flow cytometry of cells co-stained with avidin-FITC and PI. The gate for the avidin-positive cells was identified based on the negative control group. The bottom histogram shows the percentages of second-wave limbal MCs from P1, P5, P9, and P13 mice in S/G2/M phases of the cell cycle. The percentages decreased after birth, significantly at P9 and P13 relative to P1 (n = 3 repeat experiments, each experiment: P1-13, 24 limbi). *P < 0.05.

Resident immune cells in peripheral non-lymphoid tissues are constantly renewed by bone-marrow precursors^6^,^7^,^8^,^9^. However, recent studies have indicated that Langerhans cells in the epidermis, macrophages in some tissues, and microglia in the brain are capable of proliferation and that this capacity plays an essential role in the development and maintenance of these tissues^28^,^29^. Therefore, we tested the possibility that MCs in the developing cornea are capable of proliferation. We found that 50~70% of corneal MCs at E14.5 were positive for the cell-proliferation marker, Ki67. However, few corneal MCs were Ki67 positive postnatally (Fig. 4A,B), indicating that corneal MC proliferation occurs primarily prior to birth. Some MCs in the limbus were also Ki67 positive, and some were in a mitotic state (Fig. 4C). To quantify MC proliferation in the limbus after birth, we prepared single-cell suspensions from limbal tissues at P1, P5, P9, and P13 and performed cell-cycle analysis of the MCs by flow cytometry. We found that approximately one-third of the MCs were in S/G2/M phase at P1, and that this fraction decreased from P1 to P13 (Fig. 4D). These results suggest that MCs in both the cornea and limbus are capable of proliferation.

### MCs were involved in limbal vasculogenesis and corneal innervation

**Figure 5:**
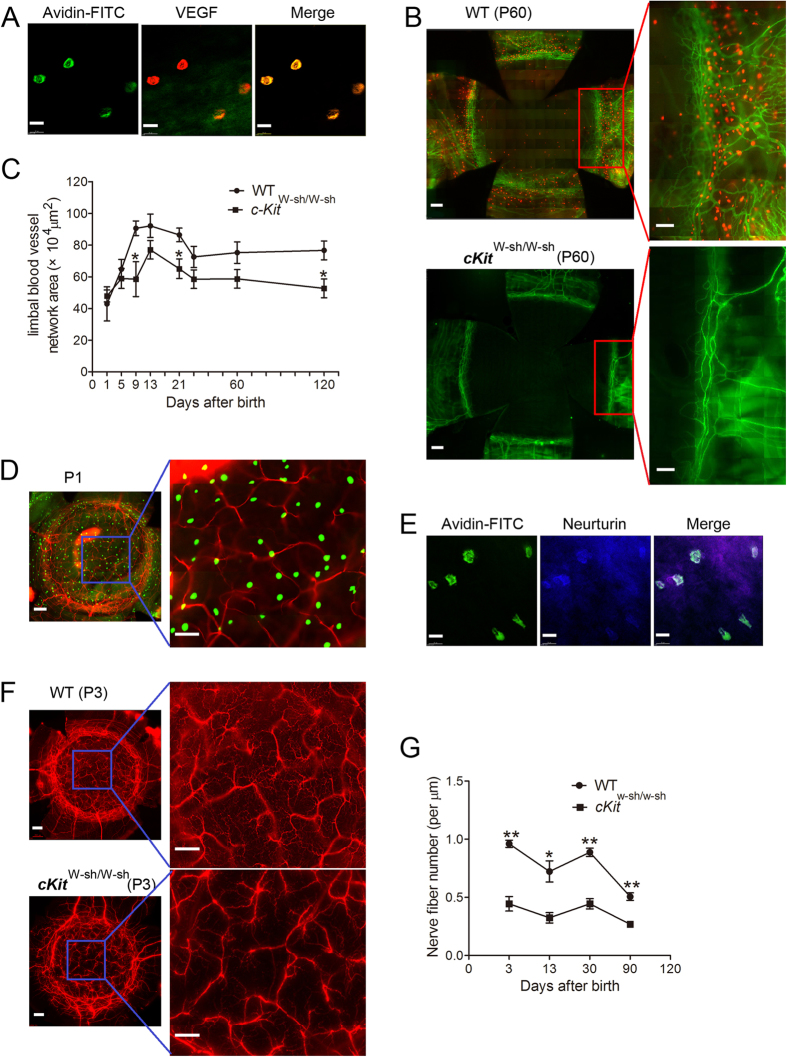
Corneal limbal blood vessel and corneal nerve fiber development in c-*Kit*^*W-sh/W-sh*^ and WT mice. (**A**) The left image shows immunostaining of the limbus from a P3 mouse with avidin-FITC (green), the middle image shows staining with VEGF-PE (red), and the right image shows these two images merged, demonstrating MCs co-staining with VEGF. Scale bars: 20 μm. (**B**) These images depict immunostaining of corneas from P60 WT and c-*Kit*^*W-sh/W-sh*^ mutant mice with anti-CD31-FITC (green) and avidin-PE (red). The c-Kit-deficient mice (lower panel) had fewer MCs and a reduced distribution of limbal blood vessels compared to the WT mice (upper panel). Scale bars: left images, 200 μm; magnified right images, 100 μm. (**C**) This graph depicts the differences in limbal vessel development in c-*Kit*^*W-sh/W-sh*^ and WT mice. From P9 to P120, the limbal vascular network area in c-Kit-deficient mice was reduced compared to that in WT mice (n = 6 corneas per age group). *P < 0.05. (**D**) Immunostaining of a P1 mouse cornea with a complete limbus with tubulin (red, a nerve marker) and avidin-FITC (green) is depicted in these images. As shown, avidin-positive cells are distributed around nerve fibers. Scale bars: left image, 200 μm; right image, 100 μm. (**E**) These images show immunostaining with avidin-FITC (green) and neurturin-ALEXA FLUOR 350 (blue) of corneas from P3 mice. The merged image shows that some MCs contained neurturin. Scale bars: 20 μm. (**F**) Tubulin staining of a cornea with a complete limbus from a P3 WT mouse and cKit-deficient mouse is shown (top images: WT, bottom images: c-*Kit*^W-sh/W-sh^). Nerve fiber density in c-*Kit*^W-sh/W-sh^ mice was reduced compared to WT mice. Scale bars: left images, 200 μm; right images, 100 μm. (**G**) The graph depicts the differences in nerve fiber density between WT and c-Kit-deficient mouse corneas. The nerve fiber density in c-*Kit*^*W-sh/W-sh*^ mouse corneas was significantly reduced compared to that in WT mice corneas at the four time points investigated (n = 6 corneas per age group). *P < 0.05, **P < 0.01.

MCs were always localized around limbal blood vessels (Supplementary Fig. 1A). We investigated whether MCs secrete vascular endothelial growth factor (VEGF) and found that they did (Fig. 5A). This close spatial relationship and the ability of MCs to produce angiogenic factors suggest that these cells may influence limbal vasculogenesis during corneal development. To test this possibility, we assessed the limbal vessel network in the MC-deficient, c-*Kit* mutant mouse line, c-*Kit*^W-sh/W-sh^. At P60, MCs could not be detected in c-*Kit*^W-sh/W-sh^ mutant mice (Fig. 5B, bottom panel). From P1 to P120, the limbal vessel network area rapidly increased after birth in WT mice, peaking from P9 to P21 and then remaining stable up to P120. In c-*Kit*^W-sh/W-sh^ mice, the limbal vessel network area was significantly reduced at P9, P21 and P120 compared with that in WT mice (Fig. 5C). These observations suggest that the impaired limbal vasculogenesis in c-*Kit*^W-sh/W-sh^ mutant mice is due to MC deficiency. Considering the spatial relationship between the blood vessels and MCs in the limbus, the second-wave MCs may play a more important role in the development of the limbal blood vessels than the first-wave MCs.

Normal innervation of the cornea is important to maintain the structure and physiological function of the cornea. MCs are localized not only around limbal vessels but also around nerve fibers in neonatal mice (Fig. 5D). They have the ability to produce nerve growth factor (NGF) and neurotrophin^30^,^31^,^32^. Our research also indicates that these MCs secrete the neurotrophin neurturin (Fig. 5E). Therefore, we inferred that these MCs were intimately involved in the development of corneal innervation. To test this hypothesis, we measured the nerve fiber density in WT and c-Kit-deficient mice during corneal development. Measurement of the corneal nerve fiber density at four postnatal time points (P3, P13, P30, and P90) revealed that the corneal nerve fiber density in c*-Kit*^W-sh/W-sh^ mice was significantly lower than that in WT mice (Fig. 5F,G). Thus, the first-wave MCs may have more chances to influence corneal innervation during development than the second-wave MCs.

### MCs did not affect the distribution of macrophages in the mouse cornea

Macrophages contribute to retinal development and, specifically, to the remodeling, vasculogenesis, and normal neuronal apoptosis that occur during retinal development. Macrophage depletion or the inhibition of their migration results in a range of developmental defects associated with the hyaloid vasculature and the pupillary membrane^33^,^34^,^35^. MCs have been identified as recruiters and regulators of leukocytes in mice^36^,^37^,^38^. Thus, MCs may function by affecting macrophage recruitment during development. To investigate this possibility, we quantified the number of F4/80-positive (F4/80^+^) macrophages in the cornea of c-*Kit*^*W-sh/W-sh*^ and WT mice during development (Supplementary Fig. 2). The F4/80^+^ macrophages increased gradually after birth, reached peak number at P11 and P12, decreased on P13, increased again, and remained stable after P21. We found that the distribution and number of the macrophages were similar between c-*Kit*^*W-sh/W-sh*^ and WT mice. These results suggest that the defects in limbal vasculogenesis and corneal nerve fibers observed in c-*Kit*^*W-sh/W-sh*^ mice are not due to a secondary loss of macrophages caused by MC deficiency.

## Discussion

The migration of mesenchymal cells into the space between the anterior epithelium of the lens vesicle and surface ectoderm is important for the formation of the corneal stroma and endothelium^2^,^3^. The function and role of other cell types in the mesenchymal stroma are less well understood. The results of this study indicate that MCs may migrate into the space adjacent to the anterior epithelium of the lens vesicle together with mesenchymal cells during the development of the cornea. We used MC-deficient animals to confirm that these cells are involved in the modulation of morphogenetic events during the development of the cornea.

MCs are conventionally thought to originate from bone marrow^6^,^7^,^8^,^9^, although Sonoda *et al.*^10^ and Hayashi *et al.*^11^ revealed that MC precursors are found in the yolk sac at E9.5 and then in fetal liver at E11. Regardless of the tissue of origin, after exiting those compartments, committed MC progenitors migrate to virtually every organ in the body. In this study, we found MCs already existed in the presumptive cornea at E12.5, which is the time of definitive hematopoiesis in fetal liver, not bone marrow^39^. This finding is in accordance with the conclusions of Sonoda *et al.*^10^ and Hayashi *et al.*^11^. Therefore, MCs found in the presumptive cornea may derive from MC precursors originating in the fetal liver or yolk sac. We then identified two waves of MC migration that occur at different developmental stages: the first wave occurs from at least E12.5 to eyelid opening (P13), and the second wave takes place from birth to P13, stabilizing after P21. The first wave involves two stages: from E12.5 to birth and from birth to eyelid opening (P13). MCs in the first stage were found mainly in the presumptive central cornea, beginning as late as E12.5. The mechanisms that underlie MC migration into the developing cornea are not yet known. Among the possible mechanisms are passive recruitment, which may occur in conjunction with mesenchymal migration, and active recruitment, which may be mediated by unidentified chemotactic molecules produced by the developing cornea. We also found that first-wave MCs in the first stage were proliferating. Thus, the gradual increase of first-wave MCs during the first stage may be caused by either continued recruitment of MC progenitors to the cornea or proliferation, or both. In contrast, the number and the proliferation capacity of these cells decreased in the second stage, when corneal MCs disappeared at P13 concurrent with eyelid opening. Additional changes in corneal development occur during the eyelid-opening period: the differentiation and proliferation of the corneal epithelium increases the thickness of the corneal epithelium and stroma, and multiple gene products show transcriptional up- or down-regulation^40^. Therefore, the decrease and disappearance of corneal MCs accompanied these changes. We also observed that, if eyelids are artificially opened at an earlier time point, the corneal MCs disappeared earlier than they did under normal eyelid opening conditions. Thus, eyelid opening induces the disappearance of MCs in the cornea.

A second wave of MCs migrated to the developing corneal limbus. These cells were located primarily around the limbal blood vessels and gradually increased in number after birth, reaching a peak at P11-12, and remained stable after P21. After birth, the bone marrow is the main site of hematopoiesis. Thus, this second wave of MCs may derive from bone marrow MC precursors. As observed with the first wave, AEO also altered the number of second-wave MCs. After AEO, the number of limbal MCs quickly increased and remained significantly higher than in controls at all time points. Recent studies have shown that MCs around blood vessels have the capacity to capture and remove IgE from the blood^41^. An increase in the number of MCs around limbal blood vessels, such as that caused by AEO, may be associated with increased susceptibility to allergic reactions. Epidemiological and experimental studies have indicated that the immunological consequences of microbial exposure during early life persist into later life^41^,^42^. One example of this phenomenon is the well-known propensity of children born prematurely to experience increased susceptibility to allergies. Our data provide some clues regarding the mechanism of this common phenomenon. In addition, our transmission electron microscopic and Alcian Blue and Safranin O staining results revealed that the first-wave comprises immature MCs, and the second-wave comprises mature MCs. These results indicate that the two types of MCs have some differences in pre-packaged granules. MCs are known to participate in various pathophysiologic processes according to their secreted granules^12^; thus, the two waves of MCs may have different roles in corneal development.

The migration of MCs to the small intestine and inflamed lung tissue relies on the adhesive molecule integrin α4β7 and the chemokine receptor CXCR2^22^,^23^,^43^,^44^,^45^. Using antibodies to these molecules, we demonstrate that the migration of second-wave MCs to the corneal limbus was also dependent on integrin α4β7 and CXCR2. The RT-PCR results revealed that expression of the α4β7 ligand VCAM1, CXCR2 ligand CXCL1, and MC growth factor SCF in the cornea was down-regulated after birth. The disappearance of first-wave MCs from the cornea might be related to the down-regulation of these molecules. Moreover, we confirmed that MCs in both the cornea and limbus are capable of proliferation. These data indicate that, as in other tissue-resident immune cells, migration and proliferation are both important for the development and homeostasis of MCs in the cornea and limbus.

A recent analysis of MCs has indicated that, beyond their participation in allergic reactions and contribution to the resistance to certain pathogens, many facets of the physiological role of MCs remain to be determined and understood^46^. Other studies have shown that these cells are also involved in the development of some organs and tissues. MCs have been found in the mammary glands of mice during postnatal development, where they participate in the rapid proliferation of mammary-gland cells and normal duct branching, independent of macrophage recruitment. The activation of serine proteases and the degranulation of MCs are necessary for the normal morphogenesis of the mammary gland^13^. MCs provide a rich source of several potent angiogenic factors, including VEGF, basic fibroblast growth factor (bFGF), transforming growth factor (TGF)-beta, tumor necrosis factor (TNF)-alpha, interleukin (IL)-8^47^,^48^,^49^,^50^,^51^, and some neurotropic factors, such as neurotrophin and NGF, which could promote and regulate the survival of nerve fibers^30^,^31^,^32^. Previous studies have shown that MCs can promote angiogenesis and nerve growth in wounds and during tumor metastasis. The relationship between these MC functions and developmental processes has not been studied extensively. We found that, during corneal development, MCs secrete VEGF and the neurotrophin neurturin and that the development of limbal blood vessels and of the corneal nerve in MC-deficient mice was markedly inhibited. Although macrophages are also important for the morphogenesis of many organs^52^, our data indicate that a deficiency of MCs did not affect the recruitment of macrophages to the cornea. Therefore, the impairment of limbal blood vessels and the corneal nerve in c-*Kit*^*W-sh/W-sh*^ mice are directly related to MC deficiency.

In conclusion, we confirmed that MCs appear in the presumptive corneal stroma at approximately E12.5. These cells may passively accompany the migration of mesenchymal cells into the cornea during development or may actively be attracted by local ocular-tissue-produced chemokines. We also found that the depletion or removal of MCs by genetic engineering led to abnormal corneal blood vessel network formation and innervation. The mechanism may be related to a deficiency in MCs locally releasing growth factors, VEGF and neurturin, or other molecules. Regardless of the mechanism, our data have uncovered another important contributor to corneal morphogenesis. Undoubtedly, further studies are required to understand which mechanisms are responsible for MC recruitment to the cornea in embryonic mice and how MCs influence the developmental process of the mouse cornea.

## Methods

### Animals

The C57BL/6 mice were obtained from Sun Yat-Sen University Laboratory Animal Center. The c-*Kit^W−sh/W−sh^* mice were purchased from The Jackson Laboratory. To examine the roles of α4β7 integrin and CXCR2 in MC-precursor homing to the cornea during eye development, three animals received an intra-peritoneal injection of anti-mouse α4β7 integrin (clone DATK32; BD, USA) and isotype IgG (clone R35-95; BD, USA), or anti-mouse CXCR2 antibody (clone 242216; R&D, USA) and isotype IgG (clone 54447; R&D, USA) (25 μg in 100 μl of saline) every other day for one week (four injections) starting at P1. To assess the effect of eyelid opening on the process of mast cell recruitment to the cornea, AEO was performed in some mice at P0 (n = 6) as described previously^53^. The eyelid fusion edge was lightly and linearly marked with the tip of a pair of scissors and pulled apart gently by hand to separate the eyelids. The contralateral eye was used as a control. All of the animal protocols were approved by the Jinan University Laboratory Animal Committee on Animal Welfare. All animals were treated in accordance with the Association for Research in Vision and Ophthalmology’s Statement for the Use of Animals in Ophthalmology and Vision Research, and the guidelines of the Animal Experimental Committee at Jinan University. Animals were anesthetized using short inhalational anesthesia with 2% isoflurane for minor procedures. Animals were euthanized by CO_2_ overdose and cervical dislocation.

### Histochemical staining

Following euthanasia, ocular tissues excised from the mice of defined ages from E12.5 to adult were fixed in 4% buffered paraformaldehyde (PFA) for 1 hour at room temperature for subsequent investigation. The ocular tissues of E12.5 mice were embedded in paraffin. The paraffin sections of E12.5 mice were stained with hematoxylin and eosin. For Toluidine Blue O staining, 0.4% (w/v) Toluidine Blue O (Cas No. 92-31-9; Sigma-Aldrich, USA) was dissolved in 60% ethyl alcohol acidified with hydrochloric acid (pH 2.0). Corneas were collected with intact limbus and fixed in 95% ethyl alcohol for 30 minutes. Next, they were washed in distilled water several times and stained with 0.4% Toluidine Blue O for 10 minutes^54^. For Alcian Blue/Safranin O staining, 1% (w/v) Alcian Blue 8GX (Cas No. 33864-99-2; Sigma-Aldrich, USA) was dissolved in 0.7 M hydrochloric acid, and 0.5% (w/v) Safranin O (Cas No. 477-73-6; Sigma-Aldrich, USA) was dissolved in 0.125 M hydrochloric acid. Corneas with complete limbus were stained in 1% Alcian Blue for 45 minutes and, after rinsing with distilled water, stained with 0.5% Safranin O for 10 minutes^54^.The stained corneas were washed several times with distilled water, cut radially, and flattened with a coverslip. All of the samples were analyzed with a DeltaVision microscopy imaging system (Applied Precision, Issaquah, WA, USA).

### Immunostaining

Immunostaining of paraffin sections: After antigen repair, the sections of ocular tissue from E12.5 mice were blocked with 2% bovine serum albumin for 10 minutes and incubated with avidin-FITC (Cas No. A-821; Life Technologies Corporation, USA) to label the MCs. Whole-mount immunofluorescence staining was performed as described previously^55^,^56^. Corneas with intact limbus were blocked with 2% bovine serum albumin for 20 minutes, permeabilized with 0.1% Triton X-100/2% bovine serum albumin for 20 minutes, and incubated with the following labeled antibodies: avidin-FITC/PE (Cas No. A-821/A-2660; Life Technologies Corporation, USA) to label MCs, anti-mouse CD31 PE or FITC (clone: L133.1/MEC 13.3; BD, USA) to label the blood vessel endothelium, anti-mouse F4/80 PE (clone: BM8; eBioscience, USA) to label macrophages, neuron-specific beta-III Tubulin NL557 (clone: TuJ-1; R&D, USA) to label nerve fibers, anti-mouse Ki67 PE (clone: 16A8; Biolegend, USA) to label proliferating nuclear antigen, anti-mouse VEGF (Catalog: AF-493-SP; R&D, USA) for immunolocalization of VEGF, and anti-neurturin (Catalog: BS-3847 R-A350; Bioss Inc., USA) for immunolocalization of neurturin. The secondary antibody for VEGF was donkey anti-goat IgG PE (Catalog: F0107; R&D, USA). Radial cuts were made in the cornea so that it could be flattened with a coverslip. The corneas were mounted in fluorescent mounting media containing 1 μM 4′,6-diamidino-2-phenylindole (DAPI) (Cas No. 28718-90-3; Sigma-Aldrich, USA) to assess the nuclear morphology. Image analysis and quantification of the corneas were performed using a DeltaVision microscopy imaging system (Applied Precision, Issaquah, WA, USA) as described previously^55^,^57^,^58^,^59^. Staining of the corneal limbus vessels with anti-CD31 antibody was used as a reference for the limbal region.

### RT-PCR

Corneas were dissected from several mice of the indicated ages. They were placed in Buffer RZ (Code No. RK145; TIANGEN, China) and smashed with TissueRuptor (QIAGEN, Germany). Total RNA was extracted using the RNA simple Total RNA Kit (Code No. DP419; TIANGEN, China). Next, we used the ReverTra Ace qPCR RT Kit (Code No. FSQ-101; TOYOBO, Japan) to obtain cDNA from the total RNA of corneas. Finally, the relative expression of the target genes compared with glyceraldehyde 3-phosphate dehydrogenase (GAPDH) was detected using THUNDERBIRD SYBR qPCR Mix (Code No. QPS-201; TOYOBO, Japan). The PCR primers used are shown in Table 1.

### Flow cytometry analysis

Many corneal limbi were cut into pieces and incubated with 500 μl of collagenase type I (Cas No. C0130; Sigma-Aldrich, USA) at ~82 U/cornea for 1.5 hours at 37 °C^60^. The cell suspensions were filtered, washed with phosphate-buffered saline (PBS), fixed at 4 °C in 4% PFA in PBS for 1 hour, permeabilized in 0.1% Triton X-100 in PBS for 10 minutes, and incubated with avidin-FITC, CD45-APC (clone: 30-F11; eBioscience, USA) and cKit-PE-Cyanine7 (clone: 2B8; eBioscience, USA) for 30 minutes to identify the expression of CD45 and cKit in avidin-positive cells, and then were analyzed by flow cytometry (BD FACS Aria, USA).

Cell suspensions of corneal limbi from different postnatal time points were fixed at 4 °C in 4% PFA in PBS for 1 hour, permeabilized in 0.1% Triton X-100 in PBS for 10 minutes, and incubated with avidin-FITC for 30 minutes. The cells were then incubated in 20 μg/ml RNase solution at 37 °C for 30 minutes, washed with PBS, incubated in 50 μg/ml propidium iodide (PI) (Cas No. P4170; Sigma-Aldrich, USA) solution for 30 minutes, and analyzed by flow cytometry (BD FACS Aria, USA) to investigate the percentages of corneal limbal MCs in the S, G2, and M phases of the cell cycle.

### Image acquisition and quantitative analysis

Using a DeltaVision microscope, the entire corneal area with intact corneal limbal vascular network was demarcated and scanned at full thickness. Z-stack image acquisition was performed using a 40 × lens and 0.20 optical section spacing. The final, stitched images were obtained using the software projection function. All of the images were divided into the corneal limbal region and corneal region (Supplementary Fig. 1A). Avidin-positive MCs or avidin and Ki67^+^ double-positive cells in the two regions were enumerated separately.

To quantify and render the vascular network area of the limbus during eye development, all of the projected images showing only FITC-CD31-positive vessels were loaded into Photoshop CS4 software. The complete vessel area in each corneal limbus was demarcated, measured, and represented by the total number of pixels. The actual area of the total vascular network was obtained by conversion of the pixels to the original scale of the image (Supplementary Fig. 3A).

To evaluate the corneal nerve density, the Line Profile of the softWoRx under the Measure Menu of the DeltaVision system was used. The acquired 3D image file including the total area of the cornea with an intact limbus was opened, and straight lines were drawn through the corneal nerve swirl center in both the horizontal and vertical directions. The number of nerve fibers that intersected the straight lines was recorded. The Distance function under the Measure Menu was used to measure the corneal diameter. The number of nerve fibers per unit length was obtained by dividing the total number of nerve fibers by the corneal diameter (Supplementary Fig. 3B).

### Transmission electron microscopy

Corneas with complete limbus from mice of defined ages were dissected, fixed in 2.5% glutaraldehyde (Code No. 18426; Pelco, USA) for 5 hours, washed three times in phosphate buffer (5 minutes each), and fixed in 1% osmic acid (Code No. 18456; Pelco, USA,) for 10 minutes. After washing in PBS three times, the tissues were dehydrated by washing in a graded ethanol series consisting of 50%, 70%, 80%, 90%, and 100% ethanol (each grade for 10 minutes). The corneas were then incubated in a 1:1 mixture of 100% ethanol:Epon812 (Code No. 18010, Pelco, USA) for 3 hours and rinsed three times with Epon812 (1 hour each). The corneas were placed in a mold filled with Epon812. The mold containing the tissues was inserted into a vacuum chamber and placed in an oven at 60 °C for 24 hours. The tissue blocks were sliced into ultrathin sections with an ultramicrotome (LKB2088, Sweden), as required for transmission electron microscopy sample preparation^61^.

### Statistical analysis

The results are presented as the means ± SEM. For comparisons between groups, one-way analysis of variance (ANOVA) was performed followed by the Tukey’s HSD test. Statistical significance was set at P < 0.05.

## Additional Information

**How to cite this article**: Liu, J. *et al.* Mast Cells Participate in Corneal Development in Mice. *Sci. Rep.* **5**, 17569; doi: 10.1038/srep17569 (2015).

## Supplementary Material

Supplementary Information

## Figures and Tables

**Table 1:** PCR primers used in this study.

Gene Name		Primer sequence	Product length (bp)	Annealing temperature (°C)
MAdCAM1	Forward	5′-CTGAGCCCTACATCCTGACCT-3′	184	57
	Reverse	5′-GCTTCACAGAGTAGCTCCCAG-3′		
VCAM1	Forward	5′-CTCCCCTGAATACAAAACGA-3′	364	
	Reverse	5′-AATCTCCAGCCTGTAAACTG-3′		
CXCL1	Forward	5′-AGTTCAGTTCTGCTTGTTCA-3′	311	
	Reverse	5′-TATGCCCTACCAACTAGACA-3′		
CXCL2	Forward	5′-CTGGATCGTACCTGATGTG-3′	367	
	Reverse	5′-TCCGACTGCATCTATTTGTC-3′		
CXCL3	Forward	5′-ATCAGAGAAAAGCGATCCAT-3′	341	
	Reverse	5′-ACATGCATTCTTCCTACACC-3′		
CXCL5	Forward	5′-GTTTGCTTAACCGTAACTCC-3′	396	
	Reverse	5′-TAGCTATGACTTCCACCGTA-3′		
SCF	Forward	5′- TGAAGAAGACACAAACTTGGA-3′	219	
	Reverse	5′- CCATATCTCGTAGCCAACAA-3′		
GAPDH	Forward	5′-CAAGGACACTGAGCAAGAG-3′	151	
	Reverse	5′-TGCAGCGAACTTTATTGATG-3′		
