# Supplementary material for: Mast Cells Participate in Corneal Development in Mice
Source: Sci Rep. 2015 Dec 2;5:17569. doi: 10.1038/srep17569 (PMC4667177; doi:10.1038/srep17569)
Supplement: Supplementary Information [file srep17569-s1.pdf]

Mast Cells Participate in Corneal Development in Mice

Jun Liu[1], Ting Fu[2], Fang Song[1], Yunxia Xue[2], Chaoyong Xia[3], Peng Liu[1], Hanqing Wang[1], Jiajun Zhong[1], Quanrong Li[1], Jiansu Chen[2], Yangqiu Li[4], Dongqing Cai[1], Zhijie Li[2,1,5,6,*]

[1]Key Laboratory for Regenerative Medicine, Jinan University, Guangzhou, China; [2]International Ocular Surface Research Center and Institute of Ophthalmology, Jinan University, Guangzhou, China; [3]Department of Embryology and Histology, Jinan University, Guangzhou, China; [4]Institute of Hematology, Jinan University, Guangzhou, China; [5]Department of Ophthalmology, First Affiliated Hospital of Jinan University, Guangzhou, China; [6]Section of Leukocyte Biology, Department of Pediatrics, Children's Nutrition Research Center, Baylor College of Medicine, Houston, Texas.

[*]Corresponding author; email address: zhijiel@bcm.edu

Supplementary information

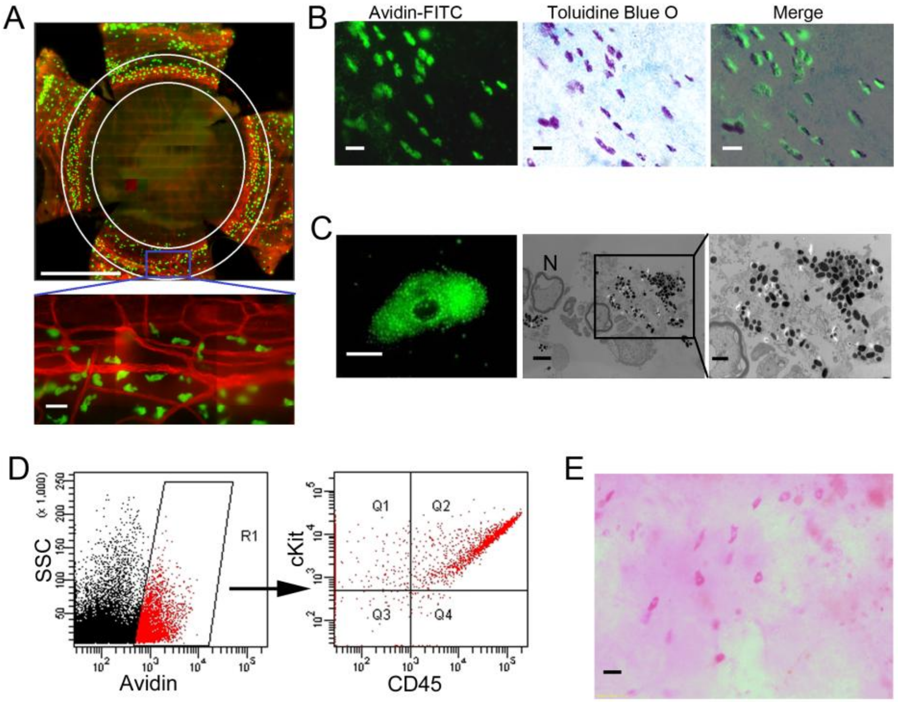

Supplementary figure 1. Identification of MCs in the corneal limbus of C57BL/6 mice. (A) This panel shows whole-mount immunofluorescence staining of corneas from mice at P60. MCs were stained with avidin-FITC (green), and blood vessels were stained with anti-CD31 PE (red). The space outside the outer circle is the conjunctiva and sclera, between the inner and outer circles

is the limbus, and the area within the inner circle is the cornea. The locally enlarged image on the right shows the distribution of detailed mast cell distribution surrounding blood vessels in the limbus. Scale bars: upper image, 1200 μm; bottom image 30 μm. (B) The corneal limbus was stained with avidin-FITC (left image) and Toluidine Blue O (center image), and the right image shows these two images merged, demonstrating the co-localization of avidin-positive and Toluidine Blue O-stained cells. Scale bars: 20 μm. (C) These panels contain images from high-resolution microscopy of MCs in the limbus of a P60 mouse. The left panel shows light microscopy of avidin-FITC staining. Scale bar: 10μm. Transmission electron microscopy of uranyl acetate staining is depicted in the center and right images. N, nerve. Scale bars: center and right, 2μm. (D) Flow cytometry analysis of avidin-positive cells. Avidin-positive cells are both CD45 and cKit positive. The left panel is the gate of avidin-positive cells (R1). The right panel is the analysis of CD45 and cKit expression in avidin-positive cells from the R1 gate. Cells in the Q2 gate are both CD45 and cKit positive. (E) This panel shows Alcian Blue–Safranin O staining of MCs in the limbus of a P60 mouse. MCs were stained only by Safranin O in red. Scale bar: 20 μm.

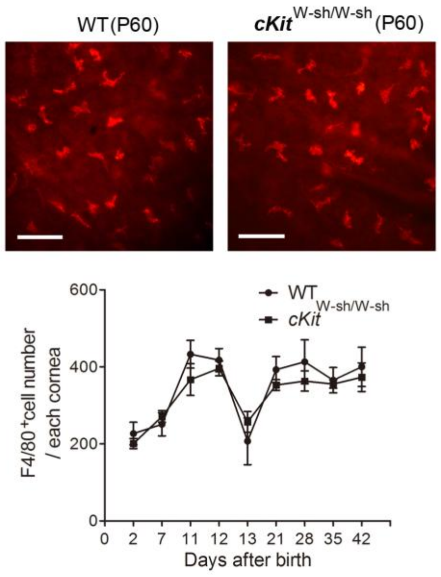

Supplementary figure 2. Distribution of macrophages in WT and c-*Kit*$^{W\text{-}sh/W\text{-}sh}$ mouse corneas. The upper images show immunostaining of macrophages in the corneas of P60 WT and c-*Kit*$^{W\text{-}sh/W\text{-}sh}$ mice with anti-mouse F4/80-PE (red) antibody. Scale bars: 50 µm. The graph depicts dynamic changes of F4/80-positive cells in the corneas of P60 WT and c-*Kit*$^{W\text{-}sh/W\text{-}sh}$ mice during development. There are no differences in the distribution of macrophages between the WT and mutant mice (n = 6 corneas per age group).

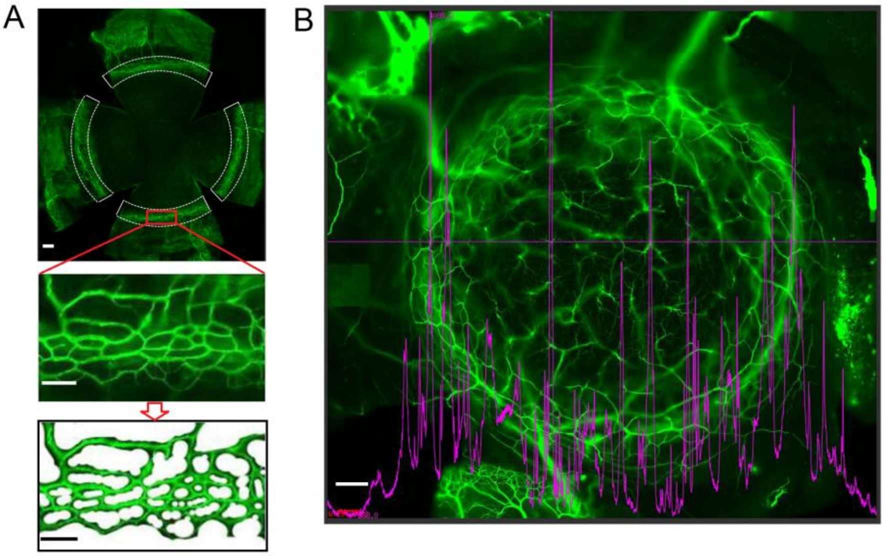

Supplementary figure 3. Calculation of the limbal blood vessel network area and corneal nerve fiber density. (A) Calculation of the limbal blood vessel network area. The upper image depicts the pattern of the limbal blood vessel network area stiched with DeltaVision software. The areas within the white dotted line are the limbal blood vessel network. The bottom image is the partial blood vessel network in the limbus after removing the redundant part. Using Photoshop CS4 software, the total number of pixels of the limbal blood vessel network area were recorded. The actual area of the total limbal blood vessel network can be obtained by conversion of the pixels to the original scale of the image. Scale bars: upper image, 200 µm; center and bottom images: 100 µm. (B) Calculation of corneal nerve fiber density using softWoRx. Each wave

represents an intersection between the line over the corneal nerve swirl center and nerve fibers. The corneal diameter was obtained using the distance function under the Measure Menu of softWoRx. Next, the number of nerve fibers per unit length was obtained by dividing the total intersected number of nerve fibers by the corneal diameter. Scale bar: 200 μm.
